# Automated Detection of Off-Label Drug Use

**DOI:** 10.1371/journal.pone.0089324

**Published:** 2014-02-19

**Authors:** Kenneth Jung, Paea LePendu, William S. Chen, Srinivasan V. Iyer, Ben Readhead, Joel T. Dudley, Nigam H. Shah

**Affiliations:** 1 Program In Biomedical Informatics, Stanford University, Stanford, California, United States of America; 2 Icahn School of Medicine at Mount Sinai, New York, New York, United States of America; 3 Center for Biomedical Informatics Research, Stanford University, Stanford, California, United States of America; University of Vermont, United States of America

## Abstract

Off-label drug use, defined as use of a drug in a manner that deviates from its approved use defined by the drug's FDA label, is problematic because such uses have not been evaluated for safety and efficacy. Studies estimate that 21% of prescriptions are off-label, and only 27% of those have evidence of safety and efficacy. We describe a data-mining approach for systematically identifying off-label usages using features derived from free text clinical notes and features extracted from two databases on known usage (Medi-Span and DrugBank). We trained a highly accurate predictive model that detects novel off-label uses among 1,602 unique drugs and 1,472 unique indications. We validated 403 predicted uses across independent data sources. Finally, we prioritize well-supported novel usages for further investigation on the basis of drug safety and cost.

## Introduction

Off-label drug use occurs when a drug is used in a manner that differs from its approved use as described by its FDA label. This practice is common and provides a pathway for clinical innovation. However, such uses escape the scientific scrutiny that goes into the labeling and marketing of new medicines [1], [2]. Estimates from office-based practices found that 21% of prescriptions are off-label [3]. Of these, 73% had little or no scientific support [3], [4], raising concerns about patient safety and costs to the healthcare system. For instance, tiagabine was approved for use as an adjunctive therapy for partial epilepsies. However, when used as the sole or primary treatment, it was found to cause seizures. In 1998, 20% of uses of tiagabine were off-label, but by 2004 this fraction had increased to 94% [5].

Off-label use is to some extent inevitable because not every condition can be tested during pre-approval [6], [7]. Nevertheless, all stakeholders in the health care system have an interest in the timely, systematic detection of off-label use. Drug manufacturers are required to report on off-label use observed in post-marketing surveillance in the European Union [8]. Regulatory agencies and clinical researchers can use knowledge of emerging off-label uses to identify potential benefits or risks that require further investigation. Furthermore, patients and their health care providers should minimize exposure to risks without clinical benefit. Unfortunately, current pharmacovigilance and post-market surveillance efforts in the United States do not monitor off-label use. Standard surveillance approaches using the FDA's Adverse Event Reporting System (FAERS) do not specifically account for use in off-label indications; efforts such as the Observational Medical Outcomes Partnership (OMOP) and the Mini-Sentinel projects do not specifically look at off-label use [9]; and physician surveys, such as the NDTI, are limited by coverage, timeliness and cost.

In this work, we focus on the problem of automatically discovering off-label uses of drugs—defined as the use of drugs for unapproved indications—from electronic health records and rank the newly discovered uses for follow up based on risk and cost metrics. At its core, we need to match drugs to the diseases they are being used to treat. We refer to such matches as drug-indication usage pairs, and say that a used-to-treat relationship exists between the drug and disease (the indication).

Previous work by Wei et al [10] used structured and semi-structured data from RxNorm, MedlinePlus, SIDER 2, and Wikipedia to compile a comprehensive list of drug-indication usage pairs. Similarly, Xu et al [11] used data from ClinicalTrials.gov and Medline to compile such a list. However, both these efforts rely on curated data sources that may not reflect current clinical practice. In contrast, the data in electronic health records represents current clinical practice and can discover such usages before they are incorporated into curated data sources.

Thus, widespread adoption of electronic medical records (EMR) provides an opportunity to detect off-label use in an automated, scalable and timely manner [8]. However, structured data in EMRs usually do not explicitly link diseases to the drugs being used to treat them [2] and is not as comprehensive as the free text of clinical notes [12]. Therefore, Natural Language Processing (NLP) is often used to extract used-to-treat relationships between drugs and indications from clinical text. Previous efforts use one of two approaches: the first approach identifies used-to-treat relationships at the level of specific occurrences of drugs and indications in text. For example, from the phrase, “on Plavix for PAD”, a used-to-treat relationship between clopidogrel and peripheral artery disease is detected. Submissions to the 2010 i2b2 NLP Challenge [13] represent the state of the art of this approach. The best performing methods require examples of text in which occurrences of drugs, indications and the relationships between them are explicitly labeled [14]. Such labeled training data is difficult to obtain (the i2b2 Challenge included 871 labeled notes) and collections of labeled text covering all drugs and indications are not available. To overcome this limitation, an alternative approach is to infer used-to-treat relationships at the population level—rather than asking whether a sentence or note implies an instance of a used-to-treat relationship, we ask whether the data as a whole suggests that a used-to-treat relationship holds in general [15]–[17]. The basic idea is to count the number of times a drug and indication are mentioned in the same clinical record, and compare that count to the expected co-mentions by chance. We have previously used such an approach for detecting drug-related adverse events [18], identifying drug-drug interactions [19], and profiling drug usages [17]. Such approaches can use relatively simple, methods for detecting drug and indication mentions in free text that do not require labeled text corpora for training. As a result, such approaches scale to very large collections of clinical text and the entire range of drugs and indications encountered in the data. In Jung et al [20], we demonstrated that it is possible to detect off-label usage using inputs derived from clinical text, combined with prior knowledge of drugs and indications from Medi-Span and DrugBank. Other researchers [21] have also used prior knowledge of known usages to match drugs and known indication mentions in clinical notes demonstrating that use of prior knowledge does improve the accuracy of detecting used-to-treat relationships.

In this paper, we build on our previous work. First, we have improved the accuracy of the classifier by taking known usage into account when counting co-mentions of drugs and indications in the clinical notes in order to reduce spurious associations arising from co-morbidities. Second, we have filtered the set of predicted novel off-label usages for support in independent, complementary data sources. We also filtered out spurious associations due to causal relationships using the SIDER 2 database [22]. Finally, in order to triage the off-label uses for follow-up, we developed indices of drug cost and risk associated with a drug's usage based on the unit price and known adverse events of drugs. These indices were used to rank off-label usages by the risk that they present to patients, along with their monetary cost. High cost and high risk usages are natural candidates for further investigation as they represent expensive and potentially dangerous cases. Whereas, low cost and low risk usages could be potential expanded indications. Our methods do not require labeled training text, and thus combine the scalability of association-based approaches with the discriminative power of machine learning techniques.

## Results

**Figure 1:**
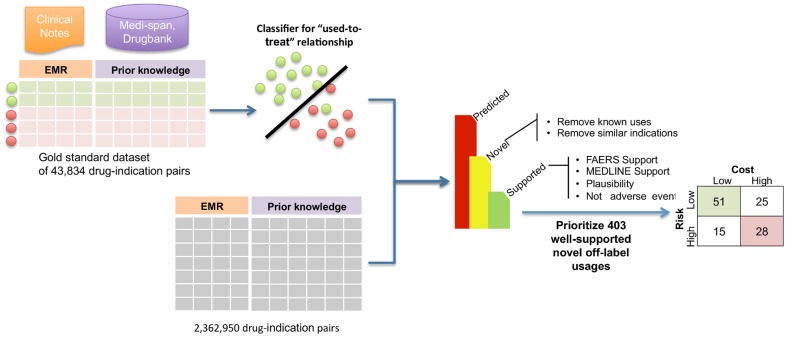
Overview of methods and results. For each of the 2,362,950 possible drug-indication pairs, we calculated 9 empirical features (e.g., co-mention count) from the free text of clinical notes in STRIDE and 16 domain knowledge features (e.g., similarity in known usage to other drugs used to treat the indication) from Medi-Span and Drugbank. These features were used by an SVM classifier trained on a gold standard dataset to recognize the used-to-treat relationship, yielding a set of predictions that were filtered for known usages, near misses in the indications, and support in two independent and complementary datasets (FAERS and MEDLINE). Predicted usages that appeared to be drug adverse events listed in SIDER 2 were removed. The resulting set of 403 well-supported novel off-label usages were binned using indices of risk and cost.

We trained an SVM classifier to recognize used-to-treat relationships between drugs and indications and applied the classifier to all possible drug-indication pairs. Filtering for high prediction confidence yielded 14,174 high confidence used-to-treat relationships. We then removed known usages listed in two curated sources of known usage — Medi-Span and the National Drug File – Reference Terminology (NDF-RT) [23], leaving 6,142 predictions that could be novel off-label usages. We assessed support for the putative novel off-label uses in independent and complementary data sources including the FDA's Adverse Event Reporting System (FAERS) and MEDLINE. When possible, we also assessed the biological plausibility of these usages using publically available gene expression data [24]. We reduce spurious results arising from drug adverse events by filtering these usages using SIDER 2, yielding a final set of 403 well-supported novel off-label usages. Overall, we tested 1,602 unique drugs and 1,475 unique indications, resulting in 403 well-supported novel off-label usages that we prioritized by their potential risks and cost. The overall approach and results are summarized in Figure 1.

### A classifier for detecting used-to-treat relationships

**Figure 2:**
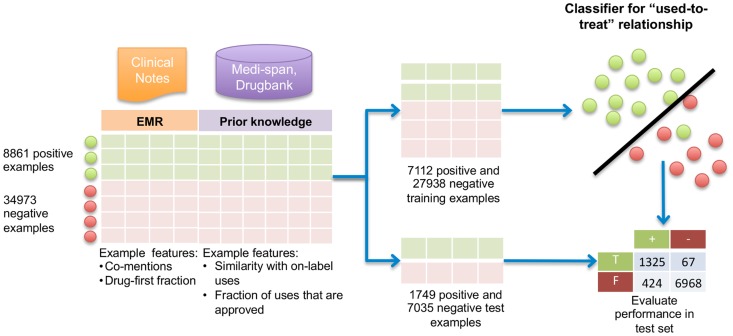
Training and testing a classifier to recognize used-to-treat relationships. We created a gold standard of positive and negative examples of known drug usage. Positive examples were taken from Medi-Span. We created negative examples by randomly selecting positive examples and then randomly choosing a drug and indication with roughly the same frequency of mentions in STRIDE as the real usage. These were then checked against Medi-Span to filter out inadvertently generated known usages. The gold standard dataset contained 4 negative examples for each positive case. For each drug-indication pair in the gold standard, we calculated features summarizing the pattern of mentions of the drugs and indications in 9.5 million clinical notes from STRIDE. We used Medi-Span and Drugbank to calculate features summarizing domain knowledge about drugs and their usages. 80% of the gold standard was used to train an SVM classifier, and the resulting model was tested on the remaining 20%.

Classifiers such as support vector machines map inputs, or *features*, to outputs. In this study, the inputs come from clinical text and domain knowledge about drugs from Medi-Span and DrugBank. Medi-Span encodes information about know usages, while Drugbank encodes information about drug targets and mechanisms of action. For each drug–indication pair, we construct a set of features that the classifier uses to predict whether a used-to-treat relationship holds between the drug and indication. The classifier learns to make accurate predictions using inputs for which we know the desired output, i.e., positive or negative examples of known usages [25]. We constructed such a *gold standard* dataset of known usages from the Medi-Span Drug Indications Database (Wolters Kluwer Health, Indianapolis, IN) as positive examples, along with negative examples constructed as detailed in Methods. An SVM classifier was trained on a random subset (80%) of the gold standard and achieved a positive predictive value of 0.963, specificity of 0.991, sensitivity of 0.764 and F1 score of 0.852 on the remaining 20% of the gold standard (see Figure 2). Feature ablation experiments showed that each group of features contributed to overall performance, particularly with respect to sensitivity and positive predictive value (Table 1). Individually, the features learned from clinical notes in the Stanford Translational Research Integrated Data Environment (STRIDE) and Medi-Span yielded sensitivities of 0.681 and 0.662 respectively, while all features together resulted in a sensitivity of 0.764.

**Table 1:** Performance of classifier on hold-out test set using different feature sets.

Feature Set	PPV	Specificity	Sensitivity	F1
Naïve STRIDE only	0.771	0.964	0.483	0.594
STRIDE only	0.936	0.988	0.681	0.788
Medi-Span only	0.945	0.990	0.692	0.778
Drugbank only	0.831	0.981	0.377	0.518
STRIDE + Medi-Span	0.967	0.994	0.744	0.841
STRIDE + Drugbank	0.936	0.988	0.697	0.799
All	0.963	0.993	0.764	0.852

We performed feature ablation experiments to assess the contribution of different feature sets to the performance of the classifier for detecting used-to-treat relationships. The first column indicates the features used to train and test the classifiers. Classifier performance was evaluated in a hold out test set of 1,749 positive and 7,035 negative examples of drug usage after training in a set of 7,112 positive and 27,938 negative examples. The first row shows performance using STRIDE derived features in which co-mentions are counted without regard to present known indications in the clinical record.

In identifying population level associations, drugs and diseases may also get associated because of causal relationships (i.e., the drug is causing the disease, as an adverse drug event) or indirect relationships (i.e., the disease is a common co-morbidity of an approved indication) rather than used-to-treat relationships. We count co-mentions of drugs and indications taking known indications into account, and as a result, obtain substantially better performance than previous methods that ignore known indications [20]. Similarly, the PPV achieved using all features was 0.963, substantially better than the 0.936 achieved using only features derived from just STRIDE and consistent with the hypothesis that prior knowledge is able to reduce spurious results arising from causal and indirect relationships [21].

### Predicting novel off-label usages

We applied an SVM trained on the entire gold standard dataset to all 2,362,950 possible drug-disease pairs to find used-to-treat relationships. SVMs do not output class membership probabilities; thus we fit a logistic regression model to the output of the SVM to estimate the probability of the used-to-treat relationship being true for a given drug-disease pair [26]. Applying a cut-off of 0.99 to this estimate yielded 14,174 high confidence used-to-treat relationships, which we interpret as potential drug-indication usage pairs. After filtering out known usages listed in Medi-Span and the National Drug File – Reference Terminology (NDF-RT) [23], we removed usages in which the predicted indication is closely related to already known indications as described in Methods, resulting in 6,142 high confidence novel usages. Because approved usages are presumably known, these are interpreted to be high confidence novel off-label usages.

### Support in FAERS, MEDLINE and SIDER 2

The 6,142 high confidence novel off-label usages were examined for positive support in two independent and complementary data sources (FAERS and MEDLINE) and for negative support in SIDER 2 as described in Methods. FAERS case reports explicitly link indications and the drugs used to treat them [27]. These reports are created by patients, health care providers and drug manufacturers, and directly reflect clinical practice. In contrast, MEDLINE provides curated annotations of the biomedical literature with terms from the National Library of Medicine's Medical Subject Headings (MeSH) vocabulary. We found that 766 novel off-label usages are supported by at least 10 records in FAERS, and 537 of those are also supported by at least two articles co-annotated with the drug and indication in MEDLINE [28]. We then filtered out usages that appeared to be bona fide drug adverse events listed in SIDER 2 in order to eliminate drug-disease pairs that are actually drug-adverse event relationships, leaving us with 466 candidate novel off-label usages. We manually examined these to filter out known usages that were missed in Medi-Span and the NDF-RT, leaving us with 403 well-supported novel off-label usages.

**Figure 3:**
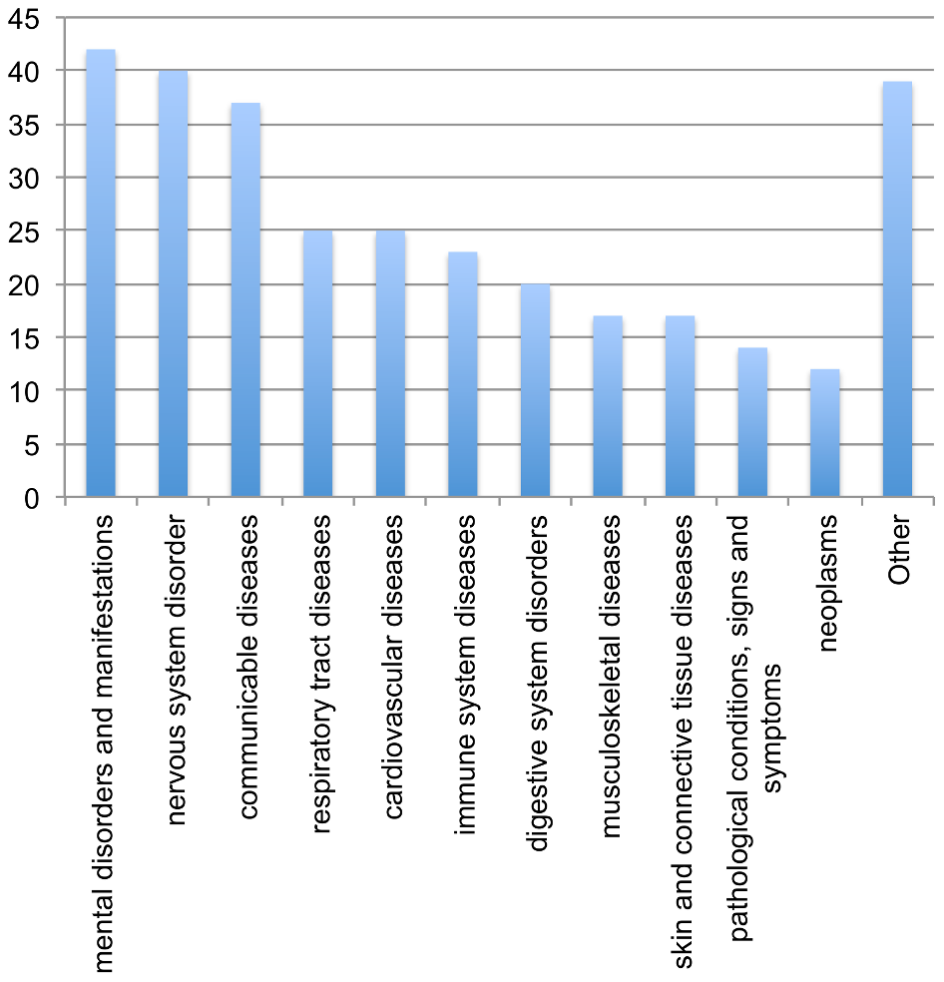
Distribution of indication classes in predicted novel usages. Each indication for the 403 high confidence novel usages with support in FAERS and MEDLINE was mapped to the first level of the NDF-RT disease hierarchy. 63 usages were not mapped to NDF-RT and were left out of this chart.

These usages (Table S1) cover 210 drugs and 184 indications, and recapitulate previously noted patterns of off-label usage (Figure 3). Medical specialties such as oncology have been noted to have high rates of off-label usage [29], [30]. Consistent with this observation, there are many cancer drugs among our results — e.g., ofatumumab for non-Hodgkin's lymphoma [31] and fludarabine for chronic myelogenous leukemia [32]. Other previously noted usage patterns include the use of the anti-seizure medications such as pregabalin and lamotrigine for migraines [33], [34], and the use of immuno-modulators such as etanercept and adalimumab, two Tumor Necrosis Factor (TNF) inhibitors, for systemic lupus erythematosus (SLE) [35], [36]. Interestingly, etanercept and infliximab, another TNF inhibitor, have both been investigated as treatments for SLE [37], lending support to the classifier's prediction. However, etanercept and adalimumab have also been implicated in causing SLE [38], [39]. Thus, in this case both the used-to-treat and causal relationships may be true.

### Plausibility based on mechanisms of action

We also evaluated the plausibility of the novel, predicted off-label usages using previously published methods [24] applied to gene expression data from the Connectivity Map [40] and NCBI Gene Expression Omnibus [41]. Briefly, if a drug modulates gene expression in the opposite manner than a disease condition, the drug is considered a plausible treatment for the indication. This approach requires gene expression data for both drug exposure and the disease condition. Of our well-supported novel usages, two had appropriate publically available data and both yielded significant gene sets suggesting possible mechanisms of action (Table S2). Given sparse coverage of drugs and diseases in public data, it is difficult to apply this process systematically. Nevertheless, this method yielded testable hypotheses regarding mechanisms of action. For instance, simvastatin is linked to diabetes by PPAR-gamma; simvastatin treatment enriches a gene set known to be activated by PPAR-gamma activity, while PPAR-gamma agonists, e.g., thiazolinediones, are known to be used to treat diabetes [42], [43].

### Manual validation of the predicted usages

Examination of the 403 well-supported novel off-label usages revealed terminological challenges. For instance, we predict that alendronic acid is used to treat osteopenia, the clinical precursor to osteoporosis. However, Medi-Span and the NDF-RT list the indication as osteoporosis instead of osteopenia — i.e., they encode the used-to-prevent relationship. Such issues reflect challenges in normalizing medical terms. As a result, although we can detect used-to-treat relationships quite well, recognizing whether or not uses are already known is difficult.

Some predicted uses represent bona fide new uses confirmed in the biomedical literature by case reports, clinical trials, or resources such as MedlinePlus, but not yet incorporated in our curated sources of known usage (see Table 2 for selected examples). For instance, our system predicts that bevacizumab is used to treat ovarian cancer. This usage has been shown to improve progression free survival in a phase III trial [44] and has been approved in the EU, but does not yet appear in Medi-Span, Drugbank, the NDF-RT or MedlinePlus. These results show that it is possible to detect emerging off-label use before it has been officially recognized.

**Table 2:** Selected predicted novel off-label usages.

Drug	Indication	FAERS Support	MEDLINE Support
Simvastatin	diabetes mellitus	1369	33
Tacrolimus	rheumatoid arthritis	404	45
Pregabalin	migraine disorders	152	3
Etanercept	lupus erythematosus, systemic	79	5
Lamotrigine	migraine disorders	75	6
Adalimumab	lupus erythematosus, systemic	71	3
Rituximab	hodgkin disease	51	48
Daptomycin	osteomyelitis	45	20
Fludarabine	waldenstrom macroglobulinemia	39	66
Infliximab	pyoderma gangrenosum	34	68
Erlotinib	malignant neoplasm of ovary	28	16

Predicted, novel drug usages with substantial support in FAERS. FAERS Support for each drug-indication pair is the number of distinct case reports in FAERS in which the drug was explicitly listed as being used to treat the indication. A complete listing is available in Table S1.

### Prioritizing predicted off-label usages for further investigation

We designed indices of drug risk and cost using adverse event associations and unit cost data from Medi-Span to objectively triage usages for further investigation. The drug risk index is normalized to lie between 0 and 1, with a value of 0 for drugs with no adverse event associations in Medi-Span (811 out of 1,602 drugs) and 1 for drugs associated with many serious adverse events. Not surprisingly, drugs with the highest risk indices were immunosupressants, such as mycophenalate mofetil, and anti-tumor agents, such as gemtuzumab, clofarabine, bevacizumab, and fludarabine. Well-supported novel off-label usages had risk indices ranging from 0.002 for amphotericin to a maximum of 0.995 for clofarabine.

The drug cost index is based on the mean unit price for the drug in Medi-Span and is also normalized to lie between 0 and 1, with a value of 1 for the drug with the highest mean unit cost in Medi-Span. The unit cost is an imperfect measure of actual treatment cost — for instance, it may be for a quantity that is sufficient for multiple treatments. Nevertheless, the cost index provides a partial ordering that is useful for relative ranking because the drugs with the highest cost index are expensive, targeted therapies such as ranibizumab, while the drugs with low cost index values are over the counter agents such as magnesium chloride and iodine.

We used the risk and cost indices to group well-supported novel off-label usages into high risk, high cost and low risk, low cost usages, resulting in 28 and 51 usages, respectively (the top 5 usages in each group are listed in Table 3; Table S3 contains the full lists). We defined thresholds for highs and lows by looking at the distribution of the risk and cost indices for the 403 well-supported usages and choosing the upper and lower quartiles as cutoffs. For example, the upper quartile for the 403 well-supported usages had risk index value 0.828, which defines the threshold for the high-risk group. For the 403 well-supported usages, Figure 1 shows the high–high (28 drug-indication pairs) and low–low groups (51 drug-indication pairs). Many (16 of 28) of the high risk, high cost usages involved anti-tumor agents being used to treat unapproved tumor types. In contrast, the low cost, low risk usages contain many over the counter drugs such as vitamin E, as would be expected.

**Table 3:** Predicted off-label usages binned by risk and cost and ranked by support in FAERS.

Drug	Indication	FAERS Support	MEDLINE Support	Risk Index	Cost Index
High risk, high cost usages					
Docetaxel	Malignant neoplasm of prostate	604	640	0.964	0.949
Clofarabine	Leukemia, myelocytic, acute	341	37	0.995	0.869
Rituximab	Purpura, thrombocytopenic, idiopathic	259	169	0.940	0.821
Bevacizumab	Malignant neoplasm of ovary	170	89	0.991	0.879
Paclitaxel	Malignant neoplasm of stomach	122	421	0.956	0.776
Low risk, low cost usages					
Folic acid	mental depression	493	18	0.082	0.102
Methadone	depressive disorder	162	33	0.002	0.167
Folic acid	hyperlipidemia	138	10	0.082	0.102
Megestrol	carcinoma, non-small cell lung	79	4	0.002	0.238
Folic acid	diarrhea	67	10	0.082	0.102

We ranked predicted, novel off-label usages on the basis of risk and cost, as represented by our risk and cost indices for each drug. FAERS Support for each drug-indication pair is the number of distinct case reports in FAERS in which the drug was explicitly listed as being used to treat the indication. The risk index is a quantitative score that represents the expected disutility of adverse events related to the use of the drug in question, normalized to the range [0, 1] so that drugs that have a higher risk of causing serious adverse events have higher values. The cost index is based on the mean unit cost of the drug in question in Medi-Span, normalized to the range [0, 1] with more expensive drugs having a higher value.

## Discussion

Off-label usage of drugs is an important enough aspect of drug safety to warrant a full issue (May 2012) of Nature Clinical Therapeutics and Pharmacology devoted to the topic [7]. Currently the most comprehensive information about off-label drug usage is from the National Disease and Therapeutic Index (IMS Health, Plymouth Meeting, PA), which relies on periodic surveys of office-based physicians. We believe that off-label use can be learned systematically, in a data-driven manner directly from electronic medical records. Our work represents the first effort to detect novel off-label usage from clinical free text over the entire range of drugs and indications observed in the medical record. We also developed quantitative risk and cost indices as a way to prioritize the novel usages for further investigation.

In the past, NLP has been applied to the problem of detecting used-to-treat relationships between drugs and indications in clinical text. State of the art NLP approaches require training text in which drug and indication mentions are labeled, along with the relationships between them. In contrast, association based approaches that use counts of drug and indication mentions are more scalable, but limited by confounding causal and indirect relationships. We have developed an automated method for detecting novel off-label usages from clinical text that does not require training text and addresses confounding relationships by incorporating prior knowledge about drug usage. We applied this method to 1,602 drugs and 1,475 indications to identify 6,142 novel off-label usages, 403 of which are well supported by evidence in independent and complementary datasets.

Our methods have important limitations. First, our work focuses on one form of off-label use — the use of drugs to treat unapproved indications — and does not detect off-label use with respect to age, gender, dosage and contraindications. Second, co-morbidities and drug adverse events may still lead to spurious used-to-treat relationships despite our efforts to reduce their impact on our results. Third, although our method can detect used-to-treat relationships between drugs and indications with high specificity and good sensitivity, the task of recognizing whether the knowledge is already known is more difficult than might be expected. This difficulty was not due to errors in recognizing terms in clinical text but rather due to mismatches in the language used to describe indications in Medi-Span and the NDF-RT versus clinical text and FAERS. A systematic listing of such indication mismatches could identify areas in ontologies and terminologies that need improvement — and would be a data-driven way to identify portions of terminologies for review. Fourth, the risk and cost indices have some shortcomings. For instance, the cost index ignores the fact that dosage and duration of treatment for off-label usages may differ from approved uses, and our risk index does not take the dependence of adverse events on dosage, co-morbidities, and poly-pharmacy into account. Finally, in this work we have aimed to produce a list of highly confident predictions of novel off-label usages so we require corroboration of predictions in FAERS, which has much lower recall in the test set than the classifier. Thus the overall method sacrifices sensitivity for greater specificity. This is appropriate for our aim in this work, but other studies may require a different trade off between sensitivity and specificity. For instance, if we were concerned exclusively with potentially risky usages, we might not require support in FAERS and instead filter for usages involving drugs associated with known serious side effects that don’t always get reported. We note that our method can be modified for such use cases.

These limitations notwithstanding, our study is the first large-scale characterization of off-label usage using fully automated methods to combine information from clinical notes with prior knowledge and to provide a ranking of the learned usages on risk and cost. It is a step towards systematic, data-driven monitoring of off-label usage. The method has characteristics that allow it to generalize to sites beyond Stanford. First, the system does not require training text labeled with mentions of drugs and indications, and the relationships between them. Second, our method is very flexible with respect to the target drug and indication vocabulary. Third, the system is very fast — annotation of 9.5 million clinical notes takes only two hours on a single machine; constructing features, training a classifier and making predictions takes an additional few hours. It is thus conceivable to process clinical text from a large number of sites, providing a picture of off-label usage across a wide spectrum of institutions.

Most importantly, our method was able to detect usages that were documented in the biomedical literature, and in one case approved in the EU, despite not appearing in any of our curated sources of known usage. This suggests that such systems could potentially provide an automated learning system for off-label usage. Such as system could flag emerging usages before they come to the attention of the broader medical community, regulatory agencies and drug manufacturers, in much the same way that Google Flu Trends can provide an early warning of flu trends in advance of CDC data [45]. We speculate that applying our method to a wider range of clinical text from multiple sites can provide a timelier and more comprehensive picture of off-label usage than is currently possible [46], [47].

## Materials and Methods

### Constructing a gold standard

We constructed a gold standard of positive and negative examples of drug usage using known usages from Medi-Span. Medi-Span contains 13,453 drug-indication pairs comprising 1,642 unique drugs and 2,313 unique indications. Of these, 1,602 of the drugs and 1,475 of the indications occur in STRIDE at least once, yielding a set of 8,861 testable drug-indication pairs. To construct negative examples, we sampled known usages from Medi-Span with replacement and then sampled new drugs and indications that occur in the data with approximately the same frequency. For instance, given the known usage “dexamethasone for systemic lupus erythematosus”, we sample a new drug from the set of drugs that occur within ten items of dexamethasone in a list of drugs sorted by overall frequency in the data. A new indication is similarly generated from systemic lupus erythematosus. Frequency matching was done because previous work suggested that frequencies can help distinguish between drug associated adverse events and treatment relationships [48]. The “negative” pairs were filtered to remove inadvertent known usages. The final gold standard consisted of 34,974 negative and 8,861 positive examples.

### Annotation of clinical text from STRIDE

We used the NCBO Annotator on free text of 9.5 million clinical notes from STRIDE to annotate the each note with mentions of drugs and indications in terms of UMLS [49] unique concept identifiers (CUI's). Negated mentions (e.g., “MI was ruled out”) or those referring to other people (e.g., “father had a stroke”) were removed using NegEx [50] and ConText [51], respectively. Drugs were normalized to 1,602 unique active ingredients (e.g., Excedrin was rewritten into acetaminophen, aspirin and caffeine) using RxNorm [52]. Indications were normalized to the set of 1,475 indications used in Medi-Span by recursively rewriting the indication as its parents in the SNOMED CT hierarchy until we reached an indication used by Medi-Span. For instance, ‘amok’ is not in the Medi-Span target vocabulary so it is rewritten as its parent term, ‘mania.’ We note that if the mentioned indication is an ancestor of the known indication, it may be counted as a novel off-label usage later on. We consider this to be reasonable because if the detected usage is broader than the known, approved usage, it is indeed off-label provided the terms are used precisely as intended. In reality, terms are not used so precisely, so we allow for some imprecision in the usage of terms when filtering out known usages from predicted usages as described below. The clinical notes covered 1.6 million patients and spanned 18 years of data, and included all clinical notes generated for these patients at Stanford Hospital during that time.

### Feature Construction

For each patient, a drug or indication is counted as present if they appear in any of the patient's notes. They count as co-occurring if they are both mentioned in the patient's notes and there is no other indication mentioned in the record that is a known usage for the drug; all co-occurrences of known indications are also counted. Doing so ensures that a drug (e.g. Lisinopril) does not get associated with a disease (e.g. Diabetes) just because the disease is a common co-morbidity of the drug's actual indication (e.g. Hypertension). In this process, known usage is defined as appearing in either Medi-Span or NDF-RT. These counts, along with derived association measures (chi squared statistic, odds ratio and conditional probability of drug mention given indication mention), were used as features. The fraction of patients in which the drug occurs before the indication (drug first fraction) was also included, along with drug first fractions adjusted for frequency of the drugs and indications [48]. Overall, we used nine features encoding the pattern of mentions of the drugs and indications in clinical text.

**Figure 4:**
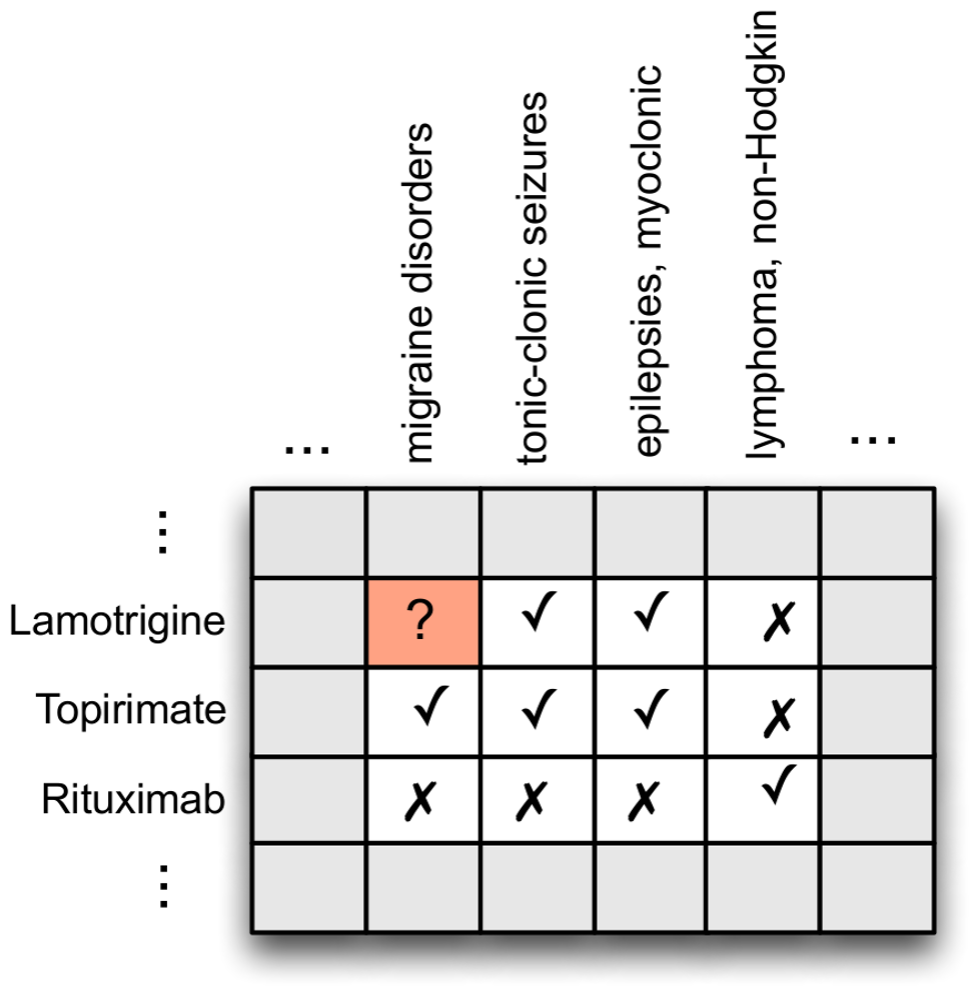
Using prior knowledge to calculate drug-drug and indication-indication similarity. We represent known usage as a matrix where row *i* represents drug *i* and column *j* represents indication *j*. A check in entry *(i,j)* indicates that the drug *i* is used to treat the indication *j*, while a cross indicates the converse. We are interested in whether a given drug, lamotrigine, is used to treat migraine disorders. We thus ask — how similar is the known usage of lamotrigine to other drugs we know are used to treat migraine disorders? Topirimate is used to treat migraine disorders, and lamotrigine is similar to it in that both are used to treat tonic-clonic seizures and myoclonic epilepsies, but not non-Hodgkin's lymphoma. This similarity in usage profile suggests that it is more likely to be used to treat migraine disorders than, say, Rituximab. We measured this similarity using the maximum cosine and Jaccard similarity of lamotrigine versus all drugs known to treat the indication. We calculate the similarity between indications based on known usage using the same data, with the roles of drugs and indications reversed.

We also used features that encode prior knowledge of the drugs, indications and known usage. These features were motivated by the intuition that drugs are typically used off-label because of some similarity with an approved drug, such as a shared molecular target, pathway or drug class [7]. We used the Medi-Span and DrugBank databases to construct features for each drug-indication pair. For Medi-Span, these included the number of drugs approved or known to be used for the indication, the fraction of known treatments for the indication that are approved, the similarity of the drug to drugs known to be used for the indication, and the similarity of the indication to other indications treated by the drug. Drug-drug similarity features were calculated as described in Figure 4. Indication-indication similarities were calculated similarly, with the role of the drugs and indications reversed. When calculating these features, we ignored known usages that were in the test set to avoid contaminating the training data with knowledge of test usages.

The DrugBank 3.0 [53] database provides information on 6,711 drugs and their molecular targets, pathways, and indications. The annotator was used to map DrugBank drug names and indications to our target sets of drugs and indications. Molecular targets, pathways, and drug categories were also extracted for each drug. We calculated similarity features analogous to the Medi-Span similarity features, along with other features that capture similarity with respect to molecular targets, pathways, and drug categories. As with the Medi-Span derived features, we removed test usages from DrugBank before calculating features. See Table S4 for a complete list of features.

### Training a predictive model

The gold standard dataset was randomly split into 35,050 training and 8,784 test examples. We trained an SVM classifier using radial basis function kernels on the training examples using the e1071 library in R. The performance of the classifier was tested on the test examples. We also trained and tested classifiers using subsets of the features to assess the contribution of different groups of features. We then trained a classifier on the entire gold standard and applied it to all 2,362,950 possible drug-indication pairs. In all cases, we used ten-fold cross validation on the training data and the “1-se” rule to select the cost hyperparameter for the SVM models. Estimates of each prediction's class membership probabilities were obtained via logistic regression [26]. We used a probability threshold of 0.99 in order to limit the set of predicted usages to the most confident predictions. This hard threshold was not tuned in any way; thus our final set of predicted novel off-label usages could possibly be improved by adjusting this threshold. However, this is a very simple way to restrict our attention to the most confident predictions.

### Identifying known usages

Known usage was determined by presence in Medi-Span or the NDF-RT – drug-indication pairs absent from both are assumed to be novel usages. Review of these usages revealed that indications were sometimes closely related to known usages — e.g., glaucoma and open angle glaucoma. We addressed this problem using biomedical ontologies, which organize biomedical concepts into hierarchies — e.g., amok is a subtype of the parent concept mania. Specifically, we removed predicted usages in which the indication is a subtype of a known usage indication in SNOMED-CT, or is the direct parent of a known usage indication in SNOMED-CT. As a final check against Medi-Span and NDF-RT, we manually reviewed predicted usages remaining after validation in FAERS, MEDLINE and SIDER2 (described below), removing 63 usages that were not detected as known usages by the methods described above.

### Validation by FAERS, MEDLINE and mechanistic plausibility

FAERS case reports contain explicit used-to-treat links between drugs and indications. We validated predicted usages using these links using public domain case reports from Q3 2007 through Q2 2012. FAERS drugs and indications were mapped to UMLS CUI's, yielding a set of 3 million drug-indication reports covering 160,989 unique pairs. Only 3,756 out of 8,861 (43%) positive examples of drug usages in our gold standard dataset appear at least once in FAERS. We required at least 10 such reports because this threshold results in a false positive rate of less than 0.005 when applied to the gold standard dataset.

MEDLINE entries are manually annotated with MeSH terms, Supplementary Concepts for drugs, and subheadings that provide further context for the annotation. For instance, an article about treatment of wet macular degeneration by bevacizumab would be annotated with “wet macular degeneration/drug therapy*” and, separately, “bevacizumab.” We downloaded the complete set of annotations for MEDLINE entries from 2002–2012. MeSH annotations were filtered for indications with the drug therapy sub-heading and mapped to UMLS CUI's using the NCBO Annotator. The Substance annotations were also mapped to UMLS CUI's using the Annotator. If no MeSH term corresponded exactly to the indication, we expanded the indication to the more general MeSH term, e.g., ‘malignant neoplasm of the ovary’ was interpreted as ‘ovarian neoplasm’. As in Avillach et al [28], we considered usages with at least three articles annotated with both the indication and the drug to be well-supported by MEDLINE.

We assessed the mechanistic plausibility of predicted usages by examining patterns of gene expression induced by the drug and indication. Briefly, we performed gene set enrichment analysis on gene expression data from the NCBI Gene Expression Omnibus (GEO) [41], [54] and the Connectivity Map [40] to identify biological pathways and expression modules that are inversely regulated between pairs of diseases and drugs, suggesting a possible basis for a therapeutic association [24]. Details of this method are in Methods S1.

### Removal of drug adverse events

We used drug adverse events listed in the SIDER 2 resource to minimize the impact of confounding causal relationships on our results. 67 out of 406 novel, well-supported off-label usages matched SIDER 2 entries. However, manual review of these matches revealed that only 28 drug-indication pairs were likely to be bona fide drug related adverse events. This is due to the fact that SIDER 2 is not curated and thus includes many spurious results such as indications being listed as adverse events. After removal of the true adverse events, 403 off-label usages remained.

### Calculation of risk and cost indices

The cost and risk indices are motivated by the observation that off-label usages do not all have the same urgency for further investigation. Decision analysis suggests that we rank usages based on their expected utility — i.e., the desirability of possible outcomes of the use, weighted by the probability each outcome [55]. For example, the use of a cheap antibiotic with few side effects to treat a rare condition has a lower urgency for follow-up than the use of an expensive drug, with severe side effects, to treat a common disorder. We approximated this approach by developing quantitative indices of drug cost and risk associated with drug usage based on known adverse events.

The cost index was calculated by ranking drugs by their mean unit cost in Medi-Span (a drug may have multiple unit costs due to different formulations, etc.). The ranks were normalized to lie between 0 and 1, with the most expensive drug having a score of 1. The risk index for each drug was based on an estimate of the *expected disutility* of adverse events associated with using that drug in Medi-Span, described in detail in Methods S1. Briefly, we assigned quantitative disutility values to adverse events associated with drugs in Medi-Span. The expected disutility of drug use due to adverse events was then estimated as the weighted sum of the disutilities for associated adverse events, with the weights given by probabilities estimated from Medi-Span's estimates of the frequency of the adverse events. Drugs were ranked by expected disutility and the ranks normalized to lie between 0 and 1 such that the riskiest drug had a value of 1. The lower and upper quartiles of the cost and risk index values observed in the 403 well-supported novel usages were used as thresholds for defining high and low risk or cost groups.

## Supporting Information

Table S1 **High confidence predicted off-label drug usages validated in FAERS and MEDLINE, with cost and risk index values.**
(PDF)

Table S2 **Molecular plausibility of off-label usages evaluated using GSEA and microarray gene expression data from GEO.**
(PDF)

Table S3 **High cost, high risk and low cost, low risk usages.**
(PDF)

Table S4 **Features used in the classifier for the** ***used-to-treat*** **relationship.**
(PDF)

Methods S1 **Additional details about methods used in this work.**
(ZIP)
