# Supplementary material for: Automated Detection of Off-Label Drug Use
Source: PLoS One. 2014 Feb 19;9(2):e89324. doi: 10.1371/journal.pone.0089324 (PMC3929699; doi:10.1371/journal.pone.0089324)
Supplement: Table S1 — High confidence predicted off-label drug usages validated in FAERS and MEDLINE, with cost and risk index values. (PDF) [file pone.0089324.s001.pdf]

Drug	Indication	FAERS Support	MEDLINE Support	Cost Index	Risk Index
venlafaxine	depressed mood	2058	95	0.481	0.857
alendronic acid	osteopenia	1996	10	0.681	0.258
mirtazapine	depressed mood	1805	89	0.399	0.932
mirtazapine	mental depression	1737	89	0.399	0.932
carboplatin	carcinoma, non-small-cell lung	1709	1126	0.821	0.485
cisplatin	carcinoma, non-small-cell lung	1599	2316	0.114	0.509
clonazepam	mental depression	1462	14	0.217	0.839
simvastatin	diabetes mellitus	1369	33	0.485	0.253
levetiracetam	seizures	1166	96	0.479	0.938
phenytoin	convulsions	1056	234	0.151	0.905
cisplatin	cancer of head and neck	668	1460	0.114	0.509
docetaxel	malignant neoplasm of prostate	604	640	0.949	0.963
rosuvastatin	diabetes mellitus	599	6	0.533	0.159
warfarin	pulmonary embolism	554	377	0.219	0.097
valproate	mental depression	552	25	0.349	0.943
capecitabine	malignant neoplasm of stomach	546	136	0.725	0.901
valproic acid	mental depression	536	25	0.350	0.941
pregabalin	diabetic neuropathies	512	53	0.482	0.833
fenofibrate	diabetes mellitus	450	17	0.469	0.656
ibandronate	osteopenia	413	8	0.902	0.070
gabapentin	fibromyalgia	389	12	0.326	0.802
clofarabine	leukemia, myelocytic, acute	341	37	0.869	0.995
risedronate	osteopenia	340	2	0.806	0.214
ezetimibe	diabetes mellitus	337	5	0.528	0.114
clopidogrel	angina pectoris	314	28	0.633	0.158
warfarin	congestive heart failure	309	62	0.219	0.097
montelukast	chronic obstructive airway disease	305	9	0.547	0.163
sertraline	bipolar disorder	278	18	0.437	0.784
simvastatin	congestive heart failure	243	23	0.485	0.253
trazodone	bipolar disorder	237	21	0.299	0.760
lorazepam	bipolar disorder	224	47	0.273	0.601
warfarin	cerebrovascular accident	220	144	0.219	0.097
risperidone	schizoaffective disorder	219	580	0.647	0.782
oxaliplatin	malignant neoplasm of stomach	204	165	0.926	0.002
simvastatin	myocardial ischemia	204	34	0.485	0.253
levofloxacin	upper respiratory infections	201	151	0.708	0.495
etoposide	hodgkin disease	198	384	0.720	0.681
allopurinol	congestive heart failure	195	22	0.572	0.289
pimecrolimus	eczema	193	15	0.454	0.103
fluoxetine	sleeplessness	190	23	0.491	0.879
citalopram	bipolar disorder	184	40	0.387	0.661
tamsulosin	malignant neoplasm of prostate	182	12	0.519	0.496
bevacizumab	malignant neoplasm of ovary	170	89	0.879	0.991
venlafaxine	bipolar disorder	170	46	0.481	0.857

paroxetine	bipolar disorder	169	42	0.426	0.870
clonazepam	migraine disorders	169	7	0.217	0.839
gemcitabine	malignant neoplasm of breast	166	335	NA	0.044
granisetron	nausea	166	90	0.808	0.515
clopidogrel	transient ischemic attack	163	58	0.633	0.158
venlafaxine	sleeplessness	154	5	0.481	0.857
scopolamine	sialorrhea	153	24	0.243	0.527
pregabalin	migraine disorders	152	3	0.482	0.833
dextromethorphan	coughing	149	76	0.078	0.198
carboplatin	cancer of head and neck	148	273	0.821	0.485
bicalutamide	benign prostatic hyperplasia	147	12	0.695	0.370
doxorubicin	burkitt lymphoma	147	130	NA	0.962
busulfan	leukemia, myelocytic, acute	145	72	0.839	0.467
lovastatin	diabetes mellitus	144	5	0.408	0.271
budesonide	bronchitis	143	19	0.783	0.698
vancomycin	systemic infection	140	152	0.726	0.002
pregabalin	restless legs syndrome	138	4	0.482	0.833
valproate	schizoaffective disorder	136	106	0.349	0.943
rosuvastatin	coronary artery disease	135	36	0.533	0.159
warfarin	angina pectoris	131	20	0.219	0.097
clarithromycin	lower respiratory tract infection	124	103	0.524	0.016
estradiol	osteoporosis	124	120	0.671	0.984
mirtazapine	schizophrenia	122	16	0.399	0.932
gemcitabine	malignant neoplasm of urinary bladde	121	281	NA	0.044
pregabalin	bipolar disorder	120	4	0.482	0.833
valproic acid	schizoaffective disorder	116	106	0.350	0.941
levetiracetam	mental depression	110	3	0.479	0.938
terbinafine	mycoses	98	31	0.635	0.341
clopidogrel	myocardial ischemia	95	60	0.633	0.158
etoposide	burkitt lymphoma	95	49	0.720	0.681
mirtazapine	bipolar disorder	93	8	0.399	0.932
carboplatin	small cell carcinoma of lung	93	45	0.821	0.485
quetiapine	obsessive-compulsive disorder	93	32	0.609	0.859
ziprasidone	schizoaffective disorder	92	110	0.654	0.002
vitamin b 12	peripheral neuropathy	90	20	0.370	0.082
memantine	mental depression	88	6	0.592	0.412
cyclosporine	behcet syndrome	87	89	0.513	0.725
telmisartan	hypercholesterolemia	86	2	0.468	0.330
tamsulosin	urinary retention	84	22	0.519	0.496
warfarin	blood coagulation disorders	81	101	0.219	0.097
tacrolimus	eczema	81	30	0.662	0.934
fluoxetine	headache	81	3	0.491	0.879
megestrol	carcinoma, non-small-cell lung	79	4	0.238	0.002
voriconazole	pneumonia	79	8	0.769	0.570
etanercept	lupus erythematosus, systemic	79	5	0.932	0.729
metformin	hyperglycemia	77	91	0.240	0.828

levofloxacin	prostatitis	75	52	0.708	0.495
lamotrigine	migraine disorders	75	6	0.534	0.899
promethazine	coughing	73	4	0.310	0.139
levetiracetam	status epilepticus	72	59	0.479	0.938
cilastatin	pneumonia	71	31	0.760	0.394
rosuvastatin	cerebrovascular accident	71	6	0.533	0.159
adalimumab	lupus erythematosus, systemic	71	3	0.959	0.615
desvenlafaxine	bipolar disorder	71	46	0.545	0.130
haloperidol	delirium	70	114	0.553	0.694
metformin	diabetes mellitus, insulin-dependent	69	73	0.240	0.828
quetiapine	personality disorders	69	5	0.609	0.859
mometasone	chronic obstructive airway disease	67	4	0.830	0.614
linezolid	systemic infection	65	24	0.825	0.277
folic acid	congestive heart failure	64	6	0.102	0.082
cisplatin	small cell carcinoma of lung	62	52	0.114	0.509
simethicone	dyspepsia	61	15	0.041	0.002
risperidone	post-traumatic stress disorder	60	19	0.647	0.782
methotrexate	inflammation	59	40	0.525	0.147
montelukast	sinusitis	59	6	0.547	0.163
pseudoephedrine	coughing	59	6	0.137	0.529
montelukast	coughing	58	9	0.547	0.163
ziprasidone	major depressive disorder	56	14	0.654	0.002
abatacept	lupus erythematosus, systemic	56	13	0.943	0.002
pravastatin	coronary artery disease	55	102	0.497	0.502
terbutaline	chronic obstructive airway disease	54	16	0.405	0.070
escitalopram	obsessive-compulsive disorder	54	76	0.537	0.002
certolizumab pegol	ulcerative colitis	52	13	0.984	0.577
rituximab	hodgkin disease	51	48	0.821	0.940
clonazepam	post-traumatic stress disorder	51	9	0.217	0.839
doxycycline	rheumatoid arthritis	51	20	0.443	0.002
folic acid	convulsions	50	10	0.102	0.082
fenofibrate	coronary artery disease	49	19	0.469	0.656
oxcarbazepine	seizures, focal	49	203	0.412	0.086
folic acid	seizures	49	21	0.102	0.082
clopidogrel	arteriosclerosis	48	50	0.633	0.158
medroxyprogesterone	menorrhagia	47	12	0.692	0.346
potassium chloride	heart failure	46	44	0.161	0.002
budesonide	sinusitis	46	18	0.783	0.698
loratadine	coughing	46	2	0.173	0.650
prednisolone	epilepsy	45	25	0.472	0.223
triazolam	anxiety disorders	45	18	0.240	0.553
daptomycin	osteomyelitis	45	20	0.913	0.002
folic acid	peripheral neuropathy	45	6	0.102	0.082
verapamil	tachycardia	44	265	0.329	0.744
loratadine	sinusitis	44	3	0.173	0.650
heparin	congestive heart failure	44	37	0.268	0.148

hydroxychloroquine	arthritis, psoriatic	43	2	0.242	0.245
clarithromycin	upper respiratory infections	43	103	0.524	0.016
escitalopram	panic disorder	42	64	0.537	0.002
anidulafungin	aspergillosis	42	25	0.874	0.002
prednisolone	angina pectoris	41	3	0.472	0.223
oxcarbazepine	trigeminal neuralgia	41	13	0.412	0.086
methimazole	graves disease	40	610	0.163	0.220
nystatin	mycoses	40	102	0.697	0.128
montelukast	rhinitis	40	14	0.547	0.163
promethazine	bronchitis	40	3	0.310	0.139
cilastatin	systemic infection	40	40	0.760	0.394
clonazepam	obsessive-compulsive disorder	40	21	0.217	0.839
simvastatin	arteriosclerosis	40	55	0.485	0.253
tacrolimus	ulcerative colitis	40	43	0.662	0.934
levofloxacin	neutropenia	39	15	0.708	0.495
levofloxacin	septicemia	39	27	0.708	0.495
fludarabine	waldenstrom macroglobulinemia	39	66	0.895	0.987
cyclophosphamide	vasculitis	39	259	0.831	0.070
medroxyprogesterone	sleep disorders	38	2	0.692	0.346
vincristine	myeloid leukemia, chronic	38	40	0.650	0.002
vigabatrin	seizures	38	52	0.779	0.002
donepezil	parkinson disease	38	36	0.635	0.410
mesalamine	ankylosing spondylitis	38	6	0.787	0.672
allopurinol	hypokalemia	38	2	0.572	0.289
leuprolide	menorrhagia	37	8	0.967	0.903
diazepam	peripheral neuropathy	37	5	0.512	0.617
levofloxacin	systemic infection	36	26	0.708	0.495
gabapentin	post-traumatic stress disorder	36	4	0.326	0.802
cilastatin	septicemia	36	40	0.760	0.394
clonazepam	major depressive disorder	36	9	0.217	0.839
sertraline	personality disorders	36	4	0.437	0.784
sucralfate	dyspepsia	35	20	0.185	0.002
mometasone	rhinitis	35	14	0.830	0.614
triptorelin	malignant neoplasm of breast	35	32	0.981	0.002
sitagliptin	hyperglycemia	35	9	0.568	0.112
flutamide	benign prostatic hyperplasia	35	30	0.348	0.321
dolasetron	nausea	34	18	0.800	0.059
triamcinolone	macular retinal edema	34	485	0.431	0.460
clarithromycin	gastroesophageal reflux disease	34	15	0.524	0.016
infliximab	pyoderma gangrenosum	34	68	0.953	0.793
nebivolol	congestive heart failure	34	61	0.356	0.068
topiramate	schizophrenia	34	21	0.522	0.846
olanzapine	post-traumatic stress disorder	34	11	0.785	0.875
mycophenolate mofetil	nephrotic syndrome	33	59	0.576	1.000
nicotine	chronic obstructive airway disease	33	4	0.221	0.227
potassium chloride	angina pectoris	33	6	0.161	0.002

prednisolone	temporal arteritis	33	154	0.472	0.223
voriconazole	systemic candidiasis	33	117	0.769	0.570
dipyridamole	hypercholesterolemia	33	10	0.313	0.595
daptomycin	bacteremia	32	89	0.913	0.002
linezolid	osteomyelitis	32	41	0.825	0.277
dapsone	acne vulgaris	32	30	0.354	0.081
amphetamine	sleep disorders	31	10	0.518	0.348
tamsulosin	prostatitis	31	13	0.519	0.496
exenatide	diabetes mellitus, insulin-dependent	31	26	0.885	0.526
alprazolam	obsessive-compulsive disorder	31	8	0.278	0.841
tinzaparin	thrombosis	30	13	0.846	0.002
levofloxacin	chronic sinusitis	30	36	0.708	0.495
mirtazapine	parkinson disease	30	3	0.399	0.932
doxazosin	malignant neoplasm of prostate	30	20	0.188	0.810
felodipine	hyperlipidemia	30	2	0.350	0.626
fenofibrate	cerebrovascular accident	29	4	0.469	0.656
amphotericin b liposoma	septicemia	29	3	0.859	0.002
heparin	hypotension	29	8	0.268	0.148
cefuroxime	upper respiratory infections	28	66	0.702	0.070
ciclesonide	chronic obstructive airway disease	28	3	0.720	0.051
erlotinib	malignant neoplasm of ovary	28	16	0.864	0.751
piperacillin	osteomyelitis	27	8	0.755	0.016
pseudoephedrine	upper respiratory infections	27	3	0.137	0.529
etanercept	inflammation	27	41	0.932	0.729
cetuximab	malignant neoplasm of larynx	27	3	0.649	0.886
adalimumab	fever	27	2	0.959	0.615
erlotinib	glioblastoma	27	28	0.864	0.751
liraglutide	diabetes mellitus, insulin-dependent	27	4	0.800	0.070
chlorpheniramine	bronchitis	26	4	0.124	0.005
nebivolol	heart failure	26	61	0.356	0.068
topotecan	neuroblastoma	26	47	0.950	0.844
guaifenesin	upper respiratory infections	26	15	0.101	0.103
mephobarbital	epilepsy	25	23	0.192	0.020
levofloxacin	bacteremia	25	43	0.708	0.495
tazobactam	osteomyelitis	25	3	0.752	0.002
pantoprazole	peptic ulcer	25	54	0.540	0.002
cyclosporine	pyoderma gangrenosum	25	84	0.513	0.725
lorazepam	muscle spasticity	24	2	0.273	0.601
methylprednisolone	migraine disorders	24	6	0.605	0.079
clonazepam	manic	24	5	0.217	0.839
tazobactam	bacterial infections	24	83	0.752	0.002
glucosamine	crohn disease	24	29	0.143	0.002
methylprednisolone	bacterial infections	23	13	0.605	0.079
sotalol	anxiety disorders	23	3	0.410	0.874
sulbactam	infection	23	2	0.699	0.097
ziprasidone	obsessive-compulsive disorder	23	5	0.654	0.002

gabapentin	seizures, focal	23	213	0.326	0.802
clotrimazole	mycoses	23	73	0.228	0.436
topiramate	post-traumatic stress disorder	23	13	0.522	0.846
paclitaxel	malignant neoplasm of urinary bladde	23	153	0.776	0.956
allopurinol	arthritis, gouty	23	45	0.572	0.289
haloperidol	alzheimer's disease	23	40	0.553	0.694
isoniazid	crohn disease	22	4	0.164	0.002
ranitidine	esophagitis	22	49	0.359	0.016
moxifloxacin	systemic infection	22	4	0.714	0.284
daptomycin	systemic infection	22	8	0.913	0.002
tazobactam	bacteremia	22	12	0.752	0.002
diclofenac	lupus erythematosus, systemic	22	5	0.409	0.756
methylprednisolone	edema	21	49	0.605	0.079
alteplase	deep vein thrombosis	21	119	0.974	0.002
prednisolone	migraine disorders	21	9	0.472	0.223
voriconazole	candidiasis	21	117	0.769	0.570
alfuzosin	urinary retention	21	24	0.517	0.104
certolizumab pegol	arthritis, psoriatic	21	3	0.984	0.577
hydrocodone	sinusitis	20	2	0.208	0.676
amphotericin b	pneumonia	20	59	0.754	0.002
methylprednisolone	angina pectoris	20	2	0.605	0.079
vancomycin	peritonitis	20	166	0.726	0.002
carbamazepine	manic	20	76	0.146	0.789
carbidopa	tremor	20	20	0.248	0.537
lamotrigine	manic	20	20	0.534	0.899
topiramate	epilepsies, myoclonic	20	11	0.522	0.846
sorafenib	gastrointestinal stromal tumors	20	8	0.815	0.919
hydrocortisone	migraine disorders	19	10	0.515	0.234
hydroxychloroquine	inflammation	19	7	0.242	0.245
methotrexate	liver diseases	19	4	0.525	0.147
methylprednisolone	pruritus	19	9	0.605	0.079
zinc oxide	hemorrhoids	19	4	0.075	0.002
pamidronate	osteogenesis imperfecta	19	110	0.835	0.971
cisplatin	malignant neoplasm of larynx	19	162	0.114	0.509
risperidone	personality disorders	19	4	0.647	0.782
topiramate	absence epilepsy	19	14	0.522	0.846
cyclophosphamide	choriocarcinoma	19	109	0.831	0.070
pimecrolimus	dermatitis	19	6	0.454	0.103
isoniazid	ulcerative colitis	18	2	0.164	0.002
tetracycline	rosacea	18	74	0.119	0.344
tramadol	major depressive disorder	18	4	0.346	0.722
zafirlukast	chronic obstructive airway disease	18	4	0.323	0.086
budesonide	bronchial spasm	18	4	0.783	0.698
cetirizine	rhinitis	18	19	0.301	0.597
nebivolol	angina pectoris	18	9	0.356	0.068
alendronic acid	hypercalcemia	18	29	0.681	0.258

pantoprazole	gastrointestinal hemorrhage	18	17	0.540	0.002
cyclosporine	acute leukemia	18	31	0.513	0.725
glucosamine	psoriasis	18	2	0.143	0.002
lovastatin	cerebrovascular accident	17	4	0.408	0.271
piroxicam	arthritis, psoriatic	17	2	0.424	0.560
prednisolone	respiratory tract infections	17	13	0.472	0.223
sulfamethoxazole	systemic infection	17	47	0.270	0.443
argatroban	deep vein thrombosis	17	4	0.941	0.002
argatroban	deep vein thrombosis	17	22	0.941	0.002
infliximab	vasculitis	17	34	0.953	0.793
etanercept	crohn disease	17	61	0.932	0.729
colchicine	hyperuricemia	17	14	0.197	0.079
factor ix	hemophilia a	17	308	0.209	0.002
imipenem	peritonitis	16	53	0.760	0.394
levofloxacin	osteomyelitis	16	32	0.708	0.495
azathioprine	lung diseases, interstitial	16	30	0.557	0.427
cefdinir	upper respiratory infections	16	9	0.442	0.002
clindamycin	crohn disease	16	3	0.516	0.548
risperidone	panic disorder	16	3	0.647	0.782
zolpidem	panic disorder	16	2	0.544	0.815
cyclophosphamide	crohn disease	16	20	0.831	0.070
cyclosporine	hepatitis, autoimmune	16	19	0.513	0.725
tacrolimus	nephrotic syndrome	16	40	0.662	0.934
micafungin	septicemia	16	2	0.877	0.507
paclitaxel	squamous cell carcinoma	16	484	0.776	0.956
methotrexate	malignant neoplasm of ovary	15	225	0.525	0.147
methylprednisolone	myositis	15	25	0.605	0.079
phenylephrine	influenza	15	3	0.130	0.262
sotalol	tachycardia	15	62	0.410	0.874
azathioprine	vasculitis	15	78	0.557	0.427
baclofen	bipolar disorder	15	8	0.367	0.712
vitamin b 12	seizures	15	4	0.370	0.082
budesonide	bronchial diseases	15	4	0.783	0.698
caspofungin	septicemia	15	6	0.929	0.002
meropenem	osteomyelitis	15	2	0.798	0.002
chlorpheniramine	upper respiratory infections	15	7	0.124	0.005
etanercept	sarcoidosis	15	15	0.932	0.729
chondroitin sulfates	rheumatoid arthritis	15	6	0.598	0.002
sirolimus	leukemia, myelocytic, acute	15	17	0.666	0.002
tazobactam	neutropenia	15	21	0.752	0.002
carboplatin	malignant neoplasm of testis	15	186	0.821	0.485
entecavir	hiv infections	15	29	0.724	0.397
drotrecogin alfa	pneumonia	15	2	0.959	0.002
olanzapine	panic disorder	15	5	0.785	0.875
fluoxetine	schizoaffective disorder	15	29	0.491	0.879
nitroglycerin	edema	15	2	0.438	0.379

haloperidol	major depressive disorder	15	22	0.553	0.694
hydroxychloroquine	dermatomyositis	14	25	0.242	0.245
amikacin	neutropenia	14	74	0.537	0.002
amikacin	infectious disease of lung	14	21	0.537	0.002
methotrexate	hodgkin disease	14	166	0.525	0.147
fexofenadine	dermatitis, atopic	14	7	0.373	0.418
vitamin b 12	convulsions	14	2	0.370	0.082
vitamin d	rickets	14	414	0.039	0.002
mirtazapine	panic disorder	14	8	0.399	0.932
captopril	cerebrovascular accident	14	6	0.209	0.454
tigecycline	systemic infection	14	6	0.824	0.628
cinacalcet	osteoporosis	14	3	0.734	0.864
estradiol	urinary incontinence	14	15	0.671	0.984
etoposide	nephroblastoma	14	34	0.720	0.681
fluconazole	aspergillosis	14	122	0.621	0.147
haloperidol	personality disorders	14	18	0.553	0.694
heparin	endocarditis	14	2	0.268	0.148
amantadine	muscle spasticity	14	3	0.191	0.198
methotrexate	multiple myeloma	13	33	0.525	0.147
pseudoephedrine	lower respiratory tract infection	13	3	0.137	0.529
levetiracetam	absence epilepsy	13	16	0.479	0.938
vancomycin	arthritis, infectious	13	22	0.726	0.002
vincristine	leukemia, myelocytic, acute	13	461	0.650	0.002
vitamin b 12	iron deficiency anemia	13	13	0.370	0.082
lansoprazole	gastrointestinal hemorrhage	13	7	0.578	0.456
leflunomide	inflammation	13	5	0.685	0.753
candesartan	migraine disorders	13	3	0.441	0.040
cilastatin	peritonitis	13	34	0.760	0.394
clonazepam	personality disorders	13	2	0.217	0.839
clotrimazole	onychomycosis	13	10	0.228	0.436
escitalopram	phobia, social	13	24	0.537	0.002
levocetirizine	dermatitis, atopic	13	2	0.439	0.408
epirubicin	carcinoma, non-small-cell lung	13	70	0.853	0.908
estradiol	polycystic ovary syndrome	13	160	0.671	0.984
sotalol	congestive heart failure	12	10	0.410	0.874
voriconazole	infectious disease of lung	12	3	0.769	0.570
cetirizine	dermatitis	12	3	0.301	0.597
finasteride	prostatitis	12	10	0.440	0.134
cefoperazone	systemic infection	12	21	NA	0.002
ondansetron	hyperemesis gravidarum	12	6	0.729	0.002
meropenem	bacteremia	12	27	0.798	0.002
alendronic acid	fracture	12	15	0.681	0.258
acyclovir	lymphoma	12	10	0.455	0.403
ranolazine	congestive heart failure	12	13	0.534	0.106
desloratadine	rhinitis	12	6	0.538	0.264
micafungin	aspergillosis	12	67	0.877	0.507

anidulafungin	candidiasis	12	69	0.874	0.002
ofatumumab	lymphoma, non-hodgkin	12	3	0.852	0.482
glucosamine	ankylosing spondylitis	12	3	0.143	0.002
ifosfamide	nephroblastoma	11	32	0.843	0.868
azathioprine	inflammation	11	25	0.557	0.427
azathioprine	liver diseases	11	46	0.557	0.427
calcitriol	kidney failure	11	4	0.558	0.002
fludarabine	hodgkin disease	11	20	0.895	0.987
fludarabine	myeloid leukemia, chronic	11	22	0.895	0.987
cephalexin	folliculitis	11	2	0.386	0.002
loratadine	dermatitis, atopic	11	10	0.173	0.650
clorazepate	bipolar disorder	11	3	0.300	0.478
chlorhexidine	multiple myeloma	11	4	0.305	0.018
cilastatin	bacteremia	11	13	0.760	0.394
oxaliplatin	malignant neoplasm of breast	11	40	0.926	0.002
clofazimine	tuberculosis	11	17	NA	0.002
cyclophosphamide	polyarteritis nodosa	11	176	0.831	0.070
glipizide	diabetes mellitus, insulin-dependent	11	8	0.166	0.044
heparin	transient ischemic attack	11	65	0.268	0.148
enoxaparin	angina pectoris	10	14	0.850	0.947
iloperidone	bipolar disorder	10	3	0.648	0.892
ranitidine	lung diseases	10	2	0.359	0.016
asparaginase	leukemia, myelocytic, acute	10	184	0.823	0.002
scopolamine	diarrhea	10	4	0.243	0.527
sulbactam	bacterial infections	10	230	0.699	0.097
azathioprine	eczema	10	11	0.557	0.427
rabeprazole	esophagitis	10	12	0.588	0.002
ibandronate	multiple myeloma	10	14	0.902	0.070
verapamil	mitral valve prolapse syndrome	10	5	0.329	0.744
azithromycin	lyme disease	10	12	0.680	0.328
clarithromycin	tuberculosis	10	41	0.524	0.016
daptomycin	arthritis, infectious	10	4	0.913	0.002
gabapentin	manic	10	15	0.326	0.802
leflunomide	lupus erythematosus, systemic	10	10	0.685	0.753
tacrolimus	dermatitis	10	23	0.662	0.934
bupropion	panic disorder	10	5	0.436	0.817
ezetimibe	hypertriglyceridemia	10	3	0.528	0.114
dextromethorphan	bronchitis	10	2	0.078	0.198
tizanidine	epilepsy	10	2	0.326	0.855
atovaquone	babesiosis	10	30	0.597	0.002
fluconazole	psoriasis	10	2	0.621	0.147
