# Supplementary material for: Automated Detection of Off-Label Drug Use
Source: PLoS One. 2014 Feb 19;9(2):e89324. doi: 10.1371/journal.pone.0089324 (PMC3929699; doi:10.1371/journal.pone.0089324)
Supplement: Table S2 — Molecular plausibility of off-label usages evaluated using GSEA and microarray gene expression data from GEO. (PDF) [file pone.0089324.s002.pdf]

| | |
|---|---|
| **Simvastatin** | **Type 2 Diabetes Mellitus** |
| **q-value <= 0.05** | |
| **Up in disease, down with treatment** | |
| AMIT SERUM RESPONSE 40 MCF10A' | 'Genes whose expression peaked at 40 min after stimulation of MCF10A cells with serum.' |
| FERRANDO T ALL WITH MLL ENL FUSION DN' | Top 100 genes negatively associated with T-cell acute lymphoblastic leukemia MLL T-ALL) expressing MLL-ENL fusion [GeneID=4297;4298].' |
| RUIZ TNC TARGETS DN' | 'Genes down-regulated in T98G cells (glioblastoma) by TNC [GeneID=3371].' |
| CROONQUIST IL6 DEPRIVATION DN' | 'Genes down-regulated in the ANBL-6 cell line (multiple myeloma, MM) after withdrawal of IL6 [GeneID=3569].' |
| **Down in disease, up with treatment** | |
| LI ADIPOGENESIS BY ACTIVATED PPARG' | 'Adipocyte genes induced in 3T3-L1 cells (adipocyte) by constitutively active PPARG [GeneID=5468] or its agonist, TZD [PubChem=5437].' |

| | |
|---|---|
| **Sirolimus** | **AML** |
| **q-value <= 0.01** | |
| **Up in disease, down with treatment** | |
| KEGG SPLICEOSOME' | 'Spliceosome' |
| MIPS SPLICEOSOME' | 'Spliceosome' |
| MIPS C COMPLEX SPLICEOSOME' | 'C complex spliceosome' |
| REACTOME PROCESSING OF CAPPED INTRON CONTAINING PRE MRNA' | 'Genes involved in Processing of Capped Intron-Containing Pre-mRNA' |
| REACTOME TRANSPORT OF MATURE TRANSCRIPT TO CYTOPLASM' | 'Genes involved in Transport of Mature Transcript to Cytoplasm' |
| REACTOME MRNA PROCESSING' | 'Genes involved in mRNA Processing' |
| REACTOME MRNA SPLICING' | 'Genes involved in mRNA Splicing' |
| REACTOME MRNA 3 END PROCESSING' | 'Genes involved in mRNA 3''-end processing' |
| REACTOME CLEAVAGE OF GROWING TRANSCRIPT IN THE TERMINATION REGION ' | 'Genes involved in Cleavage of Growing Transcript in the Termination Region ' |

| | |
|---|---|
| PICCALUGA ANGIOIMMUNOBLASTIC LYMPHOMA DN' | 'Down-regulated genes in angioimmunoblastic lymphoma (AILT) compared to normal T lymphocytes.' |
| GARY CD5 TARGETS DN' | 'Genes down-regulated in Daudi cells (B lymphocytes) stably expressing CD5 [GeneID=921] off a plasmid vector.' |
| GINESTIER BREAST CANCER 20Q13 AMPLIFICATION DN' | 'Genes down-regulated in metastatic breast cancer tumors having  type 2 amplification in the 20q13 region; involves MYBL2, STK6 and ZNF217 [GeneID=4605;6790;7764]' |
| RODRIGUES THYROID CARCINOMA POORLY DIFFERENTIATED UP' | 'Genes up-regulated in poorly differentiated thyroid carcinoma (PDTC) compared to normal thyroid tissue.' |
| RODRIGUES THYROID CARCINOMA ANAPLASTIC UP' | 'Genes up-regulated in anaplastic thyroid carcinoma (ATC) compared to normal thyroid tissue.' |
| HAHTOLA MYCOSIS FUNGOIDES CD4 DN' | 'Genes down-regulated in T helper cells (defines as CD4+) isolated from patients with mucosis fungoides compared to those from normal control donors.' |
| ENK UV RESPONSE KERATINOCYTE DN' | 'Genes down-regulated in NHEK cells (normal epidermal keratinocytes) after UVB irradiation.' |
| GRAESSMANN RESPONSE TO MC AND DOXORUBICIN DN' | 'Genes down-regulated in ME-A cells (breast cancer, sensitive to apoptotic stimuli) exposed to doxorubicin [PubChem=31703] in the presence of medium concentrate (MC) from ME-C cells (breast cancer, resistant to apoptotic stimuli).' |
| HAMAI APOPTOSIS VIA TRAIL UP' | 'Genes up-regulated in T1 cells (primary melanoma, sensitive to TRAIL [GeneID=8743]) compared to G1 cells (metastatic melanoma, resistant to TRAIL).' |
| SEIDEN ONCOGENESIS BY MET' | 'Genes changed in xenograft tumors formed by DLD-1 or DKO-4 cells (colon cancer) overexpressing MET [GeneID=4233].' |
| SCHLOSSER MYC TARGETS REPRESSED BY SERUM' | 'Cluster 7: genes up-regulated in B493-6 cells (B lymphocytes) by MYC [GeneID=4609] and down-regulated by the combination of MYC and serum.' |
| DACOSTA UV RESPONSE VIA ERCC3 COMMON DN' | 'Common down-regulated transcripts in fibroblasts expressing either XP/CS or TDD mutant forms of ERCC3 [GeneID=2071], after UVC irradiation.' |

| | |
|---|---|
| SCHAEFFER PROSTATE DEVELOPMENT AND CANCER BOX4 DN' | 'Early prostate development genes (down-regulated at 6 hr dihydrotestosterone [PubChem=10635]) which are also down-regulated in localized vs metastatic prostate cancers.' |
| SHEN SMARCA2 TARGETS UP' | 'Genes whose expression positively correlated with that of SMARCA2 [GeneID=6595] in prostate cancer samples.' |
| SCHUHMACHER MYC TARGETS UP' | 'Genes up-regulated in P493-6 cells (Burkitt''s lymphoma) induced to express MYC [GeneID=4609].' |
| PENG GLUTAMINE DEPRIVATION DN' | 'Genes down-regulated in BJAB cells (B-lymphoma) after glutamine [PubChem=738] deprivation.' |
| ZHAN MULTIPLE MYELOMA SUBGROUPS' | 'Top genes up-regulated in MM4 vs MM1 subgroup of multiple myeloma samples.' |
| MOREAUX MULTIPLE MYELOMA BY TACI DN' | 'Down-regulated genes distinguishing in multiple myeloma (MM) samples with lower expression of TACI [GeneID=23495].' |
| NOUZOVA TRETINOIN AND H4 ACETYLATION' | 'Genes whose CpG islands showed greatly increased histone H4 acetylation in NB4 cells (acute promyelocytic leukemia, APL) upon treatment with tretinoin [PubChem=5538].' |
| DEBIASI APOPTOSIS BY REOVIRUS INFECTION UP' | 'Genes up-regulated in HEK293 cells (embryonic kidney) at 6 h, 12 h or 24 h after infection with reovirus strain T3A (known as a strong inducer of apoptosis).' |
| RAMALHO STEMNESS UP' | 'Genes enriched in embryonic, neural and hematopoietic stem cells.' |
| DAZARD RESPONSE TO UV NHEK DN' | 'Genes down-regulated in NHEK cells (normal keratinocytes) by UV-B irradiation.' |
| DAZARD UV RESPONSE CLUSTER G6' | 'Cluster G6: genes increasingly down-regulated in NHEK cells (normal keratinocyte) after UV-B irradiation.' |
| DE YY1 TARGETS DN' | 'Genes down-regulated in SaOS-2 cells (osteosarcoma) upon knockdown of YY1 [GeneID=7528] by RNAi.' |
| CHEN HOXA5 TARGETS 9HR UP' | 'Genes up-regulated 9 h after induction of HoxA5 [GeneID=3205] expression in a breast cancer cell line.' |
| ZHANG TLX TARGETS 36HR DN' | 'Genes down-regulated in neural stem cells (NSC) at 36 h after cre-lox knockout of TLX (NR2E1) [GeneID=7101].' |

| | |
|---|---|
| WENDT COHESIN TARGETS UP' | 'Cohesin targets identified by ChIP-chip which were up-regulated after knockdown of CTCF and RAD21 [GeneID=10664;5885] by RNAi.' |
| MILI PSEUDOPODIA HAPTOTAXIS UP' | 'Transcripts enriched in pseudopodia of NIH/3T3 cells (fibroblast) in response to haptotactic migratory stimulus by fibronectin, FN1 [GeneID=2335].' |
| THILLAINADESAN ZNF217 TARGETS UP' | 'Genes bound and activated by ZNF217 [GeneID=7764] in MCF7 cells (breast cancer).' |
| JOHNSTONE PARVB TARGETS 2 DN' | 'Genes down-regulated upon overexpression of PARVB [GeneID=29780] in MDA-MB-231 cells (breast cancer) cultured in 3D collagen I and 3D Matrigel only.' |
| **Down in disease, up with treatment** | |
| KEGG GRAFT VERSUS HOST DISEASE' | 'Graft-versus-host disease' |
| REACTOME METABOLISM OF STEROID HORMONES AND VITAMINS A AND D' | 'Genes involved in Metabolism of steroid hormones and vitamins A and D' |
| REACTOME EXTRACELLULAR MATRIX ORGANIZATION' | 'Genes involved in Extracellular matrix organization' |
| REACTOME PEPTIDE LIGAND BINDING RECEPTORS' | 'Genes involved in Peptide ligand-binding receptors' |
| PICCALUGA ANGIOIMMUNOBLASTIC LYMPHOMA UP' | 'Up-regulated genes in angioimmunoblastic lymphoma (AILT) compared to normal T lymphocytes.' |
| SENGUPTA NASOPHARYNGEAL CARCINOMA WITH LMP1 DN' | 'Genes down-regulated in nasopharyngeal carcinoma (NPC) positive for LMP1 [GeneID=9260], a latent gene of Epstein-Barr virus (EBV).' |
| JAEGER METASTASIS DN' | 'Genes down-regulated in metastases from malignant melanoma compared to the primary tumors.' |
| TAKEDA TARGETS OF NUP98 HOXA9 FUSION 8D DN' | 'Genes down-regulated in CD34+ [GeneID=947] hematopoetic cells by expression of NUP98-HOXA9 fusion [GeneID=4928;3205] off a retroviral vector at 8 days after transduction.' |
| TAKEDA TARGETS OF NUP98 HOXA9 FUSION 10D DN' | 'Genes down-regulated in CD34+ [GeneID=947] hematopoetic cells by expression of NUP98-HOXA9 fusion [GeneID=4928;3205] off a retroviral vector at 10 days after transduction.' |

| | |
|---|---|
| TAKEDA TARGETS OF NUP98 HOXA9 FUSION 16D DN' | 'Genes down-regulated in CD34+ [GeneID=947] hematopoetic cells by expression of NUP98-HOXA9 fusion [GeneID=4928;3205] off a retroviral vector at 16 days after transduction.' |
| SABATES COLORECTAL ADENOMA UP' | 'Genes up-regulated in colorectal adenoma compared to normal mucosa samples.' |
| JAATINEN HEMATOPOIETIC STEM CELL DN' | 'Genes down-regulated in CD133+ [GeneID=8842] cells (hematopoietic stem cells, HSC) compared to the CD133- cells.' |
| CAVARD LIVER CANCER MALIGNANT VS BENIGN' | 'Genes identified by subtractive hybridization comparing malignant and benign components of a hepatocellular carcinoma (HCC) in a pre-existing liver adenoma in a morphologically normal liver.' |
| TARTE PLASMA CELL VS PLASMABLAST UP' | 'Genes up-regulated in mature plasma cells compared with plasmablastic B lymphocytes.' |
| HESS TARGETS OF HOXA9 AND MEIS1 DN' | 'Genes down-regulated in hematopoietic precursor cells conditionally expressing HOXA9 and MEIS1 [GeneID=3205;4211].' |
| LENAOUR DENDRITIC CELL MATURATION DN' | 'Genes down-regulated during in vitro maturation of CD14+ [GeneID=929] monocytes (day 0) into immature (day 7) and mature dendritic cells (day 14).' |
| LIAN LIPA TARGETS 6M' | 'Genes up-regulated at 6 months of age in lungs from LIPA [GeneID=3988] knockout mice, which display pulmonary pathology.' |
| LIAN LIPA TARGETS 3M' | 'Genes up-regulated at 3 months of age in lungs from LIPA [GeneID=3988] knockout mice, which display pulmonary pathology.' |
| MCLACHLAN DENTAL CARIES DN' | 'Genes down-regulated in pulpal tissue extracted from carious teeth.' |
| MCLACHLAN DENTAL CARIES UP' | 'Genes up-regulated in pulpal tissue extracted from carious teeth.' |
| SMID BREAST CANCER LUMINAL B DN' | 'Genes down-regulated in the luminal B subtype of breast cancer.' |
| ZHENG IL22 SIGNALING UP' | 'Genes up-regulated in ex-vivo colonic tissue after treatment with IL22 [GeneID=50616].' |
| CROMER TUMORIGENESIS UP' | 'Tumorigenesis markers of head and neck squamous cell carcinoma (HNSCC): up-regulated in the ''early'' tumors vs normal samples.' |

| | |
|---|---|
| NAKAYAMA SOFT TISSUE TUMORS PCA1 UP' | 'Top 100 probe sets contrubuting to the positive side of the 1st principal component; predominantly associated with spindle cell and pleomorphic sarcoma samples.' |
