# Supplementary material for: Automated Detection of Off-Label Drug Use
Source: PLoS One. 2014 Feb 19;9(2):e89324. doi: 10.1371/journal.pone.0089324 (PMC3929699; doi:10.1371/journal.pone.0089324)
Supplement: Table S3 — High cost, high risk and low cost, low risk usages. (PDF) [file pone.0089324.s003.pdf]

High Cost, High Risk

Drug	Indication	FAERS Support	MEDLINE Support	Cost Index	Risk Index
docetaxel	malignant neoplasm of prostate	604	640	0.949	0.963
clofarabine	leukemia, myelocytic, acute	341	37	0.869	0.995
bevacizumab	malignant neoplasm of ovary	170	89	0.879	0.991
rituximab	hodgkin disease	51	48	0.821	0.940
fludarabine	waldenstrom macroglobulinemia	39	66	0.895	0.987
leuprolide	menorrhagia	37	8	0.967	0.903
olanzapine	post-traumatic stress disorder	34	11	0.785	0.875
topotecan	neuroblastoma	26	47	0.950	0.844
paclitaxel	malignant neoplasm of urinary bladder	23	153	0.776	0.956
sorafenib	gastrointestinal stromal tumors	20	8	0.815	0.919
pamidronate	osteogenesis imperfecta	19	110	0.835	0.971
paclitaxel	squamous cell carcinoma	16	484	0.776	0.956
olanzapine	panic disorder	15	5	0.785	0.875
epirubicin	carcinoma, non-small-cell lung	13	70	0.853	0.908
ifosfamide	nephroblastoma	11	32	0.843	0.868
fludarabine	hodgkin disease	11	20	0.895	0.987
fludarabine	myeloid leukemia, chronic	11	22	0.895	0.987
enoxaparin	angina pectoris	10	14	0.850	0.947

Low Cost, Low Risk

Drug	Indication	FAERS Support	MEDLINE Support	Cost Index	Risk Index
warfarin	pulmonary embolism	554	377	0.219	0.097
warfarin	congestive heart failure	309	62	0.219	0.097
warfarin	cerebrovascular accident	220	144	0.219	0.097
dextromethorphan	coughing	149	76	0.078	0.198
warfarin	angina pectoris	131	20	0.219	0.097
warfarin	blood coagulation disorders	81	101	0.219	0.097
megestrol	carcinoma, non-small-cell lung	79	4	0.238	0.002
promethazine	coughing	73	4	0.310	0.139
folic acid	congestive heart failure	64	6	0.102	0.082
simethicone	dyspepsia	61	15	0.041	0.002
folic acid	convulsions	50	10	0.102	0.082
folic acid	seizures	49	21	0.102	0.082
potassium chloride	heart failure	46	44	0.161	0.002
folic acid	peripheral neuropathy	45	6	0.102	0.082
heparin	congestive heart failure	44	37	0.268	0.148
hydroxychloroquine	arthritis, psoriatic	43	2	0.242	0.245

methimazole	graves disease	40	610	0.163	0.220
promethazine	bronchitis	40	3	0.310	0.139
sucralfate	dyspepsia	35	20	0.185	0.002
nicotine	chronic obstructive airway disease	33	4	0.221	0.227
potassium chloride	angina pectoris	33	6	0.161	0.002
heparin	hypotension	29	8	0.268	0.148
chlorpheniramine	bronchitis	26	4	0.124	0.005
guaifenesin	upper respiratory infections	26	15	0.101	0.103
mephobarbital	epilepsy	25	23	0.192	0.020
glucosamine	crohn disease	24	29	0.143	0.002
isoniazid	crohn disease	22	4	0.164	0.002
hydroxychloroquine	inflammation	19	7	0.242	0.245
zinc oxide	hemorrhoids	19	4	0.075	0.002
isoniazid	ulcerative colitis	18	2	0.164	0.002
zafirlukast	chronic obstructive airway disease	18	4	0.323	0.086
glucosamine	psoriasis	18	2	0.143	0.002
colchicine	hyperuricemia	17	14	0.197	0.079
factor ix	hemophilia a	17	308	0.209	0.002
phenylephrine	influenza	15	3	0.130	0.262
chlorpheniramine	upper respiratory infections	15	7	0.124	0.005
hydroxychloroquine	dermatomyositis	14	25	0.242	0.245
vitamin d	rickets	14	414	0.039	0.002
heparin	endocarditis	14	2	0.268	0.148
amantadine	muscle spasticity	14	3	0.191	0.198
glucosamine	ankylosing spondylitis	12	3	0.143	0.002
chlorhexidine	multiple myeloma	11	4	0.305	0.018
glipizide	diabetes mellitus, insulin-dependent	11	8	0.166	0.044
heparin	transient ischemic attack	11	65	0.268	0.148
dextromethorphan	bronchitis	10	2	0.078	0.198
