# Supplementary material for: Automated Detection of Off-Label Drug Use
Source: PLoS One. 2014 Feb 19;9(2):e89324. doi: 10.1371/journal.pone.0089324 (PMC3929699; doi:10.1371/journal.pone.0089324)
Supplement: Table S4 — Features used in the classifier for the used-to-treat relationship. (PDF) [file pone.0089324.s004.pdf]

Features used for *used-to-treat* classifier

co.occur.counts
drug.first.fractions
loess.drug.first.fractions.fixed.drug
loess.drug.first.fractions.fixed.disorder
drug.counts
disorder.counts
odds.ratio of drug-indication co-occurrence
confidence (probability of drug occurring given the drug occurs)
chi-sq statistic for drug-indication co-occurrence
medispan.approved.drugs.for.disorder.count
medispan.known.drugs.for.disorder.count
medispan.fraction.approved.drugs.for.disorder
max.cos.sim.for.known.disorders
max.jac.sim.for.known.disorders
max.cos.sim.for.known.drugs
max.jac.sim.for.known.drugs
drugbank.max.cos.sim.for.known.disorders
drugbank.max.jac.sim.for.known.disorders
drugbank.max.cos.sim.for.known.drugs
drugbank.max.jac.sim.for.known.drugs
drugbank.max.cos.pathway.sim.for.known.drugs
drugbank.max.jac.pathway.sim.for.known.drugs
drugbank.max.cos.category.sim.for.known.drugs
drugbank.max.jac.category.sim.for.known.drugs
drugbank.has.common.target.with.known.drug
