# Supplementary material for: Automated Detection of Off-Label Drug Use
Source: PLoS One. 2014 Feb 19;9(2):e89324. doi: 10.1371/journal.pone.0089324 (PMC3929699; doi:10.1371/journal.pone.0089324)
Supplement: Methods S1 — Additional details about methods used in this work. (ZIP) [file pone.0089324.s005.zip]

Supplementary Materials – Methods
This section provides further detail about the methods used in the paper.

Molecular assessment of plausibility
We performed gene set enrichment analysis to identify biological pathways and functional groups that may be inversely regulated between pairs of diseases and drugs, thus suggesting a possible basis for a therapeutic association(1). Disease expression data was obtained from the NCBI Gene Expression Omnibus (GEO)(2, 3), using methods previously described (4, 5). The microarray series for the diseases under consideration are as follows: Bipolar Affective Disorder: GSE5338; Multiple Lyeloma: GSE5900; Acute Myeloid Leukemia: GSE2191; Type 1 Diabetes Mellitus: GSE10586; Type 2 Diabetes Mellitus: GSE2952.

Rank normalization was applied to the disease expression data in order to carry out robust cross-platform analysis(6, 7). Since experiments were run on different platforms, we standardize gene identifiers from chip specific probe identifiers to NCBI GeneID identifiers using AILUN(8). In cases where multiple microarray probes mapped to the same NCBI GeneID, we averaged across individual probe expression values.

Drug-exposure gene expression microarray data measured on several types of cancer cell lines in the context of the reported drugs was obtained from Lamb et al.(9). The experiments were carried out the breast cancer epithelial cell line MCF7, the prostate cancer epithelial cell line PC3 and the nonepithelial lines HL60 (leukemia). The original experiments were carried out on two platforms: GEO Platform (GPL) 96 Affymetrix GeneChip Human Genome U133 Array Set HG-U133A and GPL3921 Affymetrix GeneChip HT-HG_U133A Arrays. In order to be able to reason between drug and disease expression data, we standardized gene identifiers from microarray specific probe identifiers to NCBI GeneID identifiers, averaging across individual probe expression values. We followed the pre-processing and normalization steps described in the Supplemental materials in Lamb et al. (9).

The ranked gene expression lists underlying the drug signatures and the disease expression data were annotated using the GSEA pre ranked tool(10, 11), using the Molecular Signatures Database C2 collection, which comprises 4,850 curated gene sets derived from online pathway databases, PubMed publications, and domain expert knowledge(11). The GSEA pre-ranked tool looks for overrepresentation of each gene set in C2, towards the top or bottom of the ranked gene expression list, to allow inference of positive or negative enrichment of each gene set within the context of that specific gene list. An enrichment score (positive or negative) is then defined by the farthest deviation from zero, for that particular list, and then compared with the enrichment scores of 1,000 permutations (shuffling gene set members in a given gene set) to obtain nominal p-values and the q-value statistic used to associate an enriched module with a disease or drug expression in this study.

Rank-files comprising a list of gene identifiers and a score that is used to rank the gene expression level were input into the GSEA pre-ranked tool. In the case of disease

expression data, the score increments were determined by the log2 fold-change for each gene in the list, and for the drug expression data, where log2 fold-change data wasn't available, scores were created on the basis of inverse-ranks (the most over-expressed gene was associated with the highest score), which were then centered around zero, so that genes associated with the top half of the list would increment a given enrichment score positively, and the bottom half would decrement an enrichment score in a negative direction.

For each drug, all positively (and separately, negatively) enriched gene sets (with a q-value at or below 0.01/0.05 as shown in Supplementary Materials S2) associated with any instance of that compound in connectivity map, were identified. For each disease, all positively (and separately, negatively) enriched gene sets (with a q-value at or below 0.01/0.05 as shown in Supplementary Materials S2) were identified. Occurrences of intersecting, significantly enriched gene sets that are 'positively-enriched-in-disease / negatively-enriched-in-drug' (and conversely 'negatively-enriched-in-disease / positively-enriched-in-drug') were thus identified, potentially indicating biological pathways which could underlie a therapeutic association between a drug and disease.

Calculation of risk and cost indices

The cost index was calculated by ranking drugs by their mean unit cost as recorded in Medi-Span (a given drug may have multiple unit costs recorded in Medi-Span due to differing formulations, etc). These ranks were then normalized to lie between 0 and 1, such that the drug with the highest mean unit cost had a score of 1, while drugs with the lowest cost had a score of 0. The risk index for each drug was based on an estimate of the *expected disutility* of adverse events associated with using that drug in Medi-Span. We assigned a numeric value to each drug-adverse event association – the disutility – that quantifies the severity of the adverse event. We restricted drug-adverse event associations to those based on either FDA black box warnings, well controlled human studies, or multiple case reports and uncontrolled human studies. Medi-Span also assigns severity levels to the drug-adverse event associations, with each association assigned to 'Major', 'Moderate', or 'Minor'; we further classified 139 Major adverse events as 'Severe' (listed at the end of this section). These categories were assigned disutility values of 1000, 100, 10 and 1 respectively, so that severe events had higher disutility than minor adverse events. Medi-Span also provides estimates of the frequency of adverse events given drug use in discrete bins – we used probabilities of 0.1, 0.04, 0.01 and 0.001 for the most common to least common frequency bins. For each drug and its associated adverse events, we weighted the disutilities of the adverse events by their frequency estimates and summed them to obtain the expected disutility for each drug. Drugs were then ranked by their expected disutility and the ranks normalized such that the drug with the highest expected disutility had an index value of 1 while drugs with no associated adverse events had an index value of 0. This method assumes that adverse events are independent of each other, and ignores dose and other factors affecting the incidence of adverse events, but it accomplished the goal of assigning high values to drugs that are risky (e.g., mycophenolate mofetil and clofarabine) and low values to less risky drugs (e.g., acetaminophen and vitamin E). The lower and upper quartiles of the cost and risk index values observed in the 635 novel, validated usages were used as

thresholds for defining high and low risk or cost groups.

List of major adverse events
thrombosis
heart failure
ecg: ventricular tachycardia
myocardial failure
septicemia
systemic infection
death (finding)
seizure observable
diabetes mellitus
kidney tubular necrosis, acute
metabolic acidosis
malignant neoplasm of breast
cerebrovascular accident
airway obstruction
cyanosis
ecg: ventricular arrhythmia
thromboembolism
hypertensive crisis
hypertensive encephalopathy
feeling suicidal (finding)
suicidal intent
hepatic necrosis
cardiac arrest
cardiopulmonary arrest
cardiorespiratory failure
kidney failure
malignant neoplasm of skin
ecg: complete atrioventricular block
psychotic disorders
torsades de pointes
ecg: ventricular fibrillation
tonic - clonic seizures
cerebral edema
convulsions
seizures
brain edema
acute myocardial infarction
congestive heart failure
graft occlusion, vascular
status epilepticus
hypertonic contractions
neural tube defects
gastrointestinal perforation

pulmonary embolism
shock, cardiogenic
subsequent myocardial infarction
acute leukemia
leukemia, myelocytic, acute
pulmonary edema
acute myeloid leukemia without mention of remission
endometrial carcinoma
respiratory distress syndrome, adult
pulmonary insufficiency following trauma
pulmonary insufficiency following shock
pulmonary insufficiency following surgery
ventricular tachycardia, polymorphic
hemorrhagic diarrhea
cerebral hemisphere hemorrhage
intracranial hemorrhages
systemic candidiasis
asphyxia
cholecystitis
pneumothorax
pneumonia, pneumocystis carinii
anaphylaxis
liver failure
arterial aneurysm
comatose
disseminated intravascular coagulation
o/e - dead - sudden death
paralysed
intestinal perforation
o/e - loss of consciousness
suicide
liver carcinoma
kidney failure, acute
laryngeal edema
toxic epidermal necrolysis
anaphylactoid reaction
hepatic coma
neuroleptic malignant syndrome
hepatocellular adenoma
drug-induced hemolytic anemia
heparin-induced thrombocytopenia with thrombosis
rhabdomyolysis
cerebral ischemia
catatonia
suicide attempt
ventricular septal defects

mitral valve insufficiency
hepatotoxicity
narcolepsy
o/e - quadriplegia
blindness and/or vision impairment level
liver neoplasms
acute pancreatitis
hepatic encephalopathy
brain injuries
shock
gangrene
retinal degeneration
diabetic ketoacidosis
retroperitoneal hemorrhage
ecg: supraventricular arrhythmia
reye syndrome
megacolon, toxic
chest pain
vasospasm
venous thrombosis
psychotic symptom
peripheral gangrene
malignant hyperpyrexia due to anesthesia
encephalitis
hematoma, subdural
hemiplegia
cerebral arterial thrombosis
pyelonephritis
homicidal thoughts
demyelinating disease of central nervous system
leukemia
lymphoma
colitis, ischemic
deafness
left-sided heart failure
severe major depression with psychotic features
acute left ventricular failure
hearing impairment
thrombotic microangiopathies
graft-vs-host disease
spontaneous abortion
chronic active hepatitis
retinal detachment
malignant neoplasm of urinary bladder
psychoactive substance-induced organic delusional disorder
drug-induced paranoid state

o/e - resp. arrest
malignant neoplasms
acute coronary syndrome
neoplasm of unspecified nature of breast (disorder)

References

1. Sirota M, Dudley JT, Kim J, Chiang AP, Morgan AA, Sweet-Cordero A, et al. Discovery and preclinical validation of drug indications using compendia of public gene expression data. Science translational medicine. 2011 Aug 17;3(96):96ra77. PubMed PMID: 21849665. Pubmed Central PMCID: 3502016.
2. Barrett T, Edgar R. Gene expression omnibus: microarray data storage, submission, retrieval, and analysis. Methods in enzymology. 2006;411:352-69. PubMed PMID: 16939800. Pubmed Central PMCID: 1619900.
3. Edgar R, Domrachev M, Lash AE. Gene Expression Omnibus: NCBI gene expression and hybridization array data repository. Nucleic acids research. 2002 Jan 1;30(1):207-10. PubMed PMID: 11752295. Pubmed Central PMCID: 99122.
4. Dudley J, Butte AJ. Enabling integrative genomic analysis of high-impact human diseases through text mining. Pacific Symposium on Biocomputing Pacific Symposium on Biocomputing. 2008:580-91. PubMed PMID: 18229717. Pubmed Central PMCID: 2735266.
5. Dudley JT, Tibshirani R, Deshpande T, Butte AJ. Disease signatures are robust across tissues and experiments. Molecular systems biology. 2009;5:307. PubMed PMID: 19756046. Pubmed Central PMCID: 2758720.
6. Abba MC, Hu Y, Sun H, Drake JA, Gaddis S, Baggerly K, et al. Gene expression signature of estrogen receptor alpha status in breast cancer. BMC genomics. 2005;6:37. PubMed PMID: 15762987. Pubmed Central PMCID: 555753.
7. Warnat P, Eils R, Brors B. Cross-platform analysis of cancer microarray data improves gene expression based classification of phenotypes. BMC bioinformatics. 2005;6:265. PubMed PMID: 16271137. Pubmed Central PMCID: 1312314.
8. Chen R, Li L, Butte AJ. AILUN: reannotating gene expression data automatically. Nature methods. 2007 Nov;4(11):879. PubMed PMID: 17971777. Pubmed Central PMCID: 2716375.
9. Lamb J, Crawford ED, Peck D, Modell JW, Blat IC, Wrobel MJ, et al. The Connectivity Map: using gene-expression signatures to connect small molecules, genes, and disease. Science. 2006 Sep 29;313(5795):1929-35. PubMed PMID: 17008526.
10. Mootha VK, Lindgren CM, Eriksson KF, Subramanian A, Sihag S, Lehar J, et al. PGC-1alpha-responsive genes involved in oxidative phosphorylation are coordinately downregulated in human diabetes. Nature genetics. 2003 Jul;34(3):267-73. PubMed PMID: 12808457.
11. Subramanian A, Tamayo P, Mootha VK, Mukherjee S, Ebert BL, Gillette MA, et al. Gene set enrichment analysis: a knowledge-based approach for interpreting genome-wide expression profiles. Proceedings of the National Academy of Sciences of the United States of America. 2005 Oct 25;102(43):15545-50. PubMed PMID: 16199517. Pubmed Central PMCID: 1239896.
